# Our Contemporaries

**Published:** 1912-02

**Authors:** 


### OUR CONTEMPORARIES

---

THE *Zentralblatt für Chirurgie,* No. 35 of 1911, abstracts Dr. Edgar McGuire's article on Cerebral Compression, from the BUFFALO MEDICAL JOURNAL of July 1911. If any of our readers have the idea that western New York is not capable of supporting a medical journal that is worthy of respect, aside from its function as a medium of local news, let them remember that this is the sixth article (inclusive of three editorials reprinted in toto) which has been considered worth reproducing in more than passing mention, by our exchanges, in as many months. And it is perfectly possible that many other abstracts have been made which have not come to our attention.

THE *American Journal of Surgery,* January 1912, has a special "western issue," containing 16 splendid articles by as many splendid men. We cannot even list them, much less abstract the articles, but would advise all interested in surgery and borderline studies to get a copy and read it. Just to be contrary, we have averaged, approximately, the longitude of the places where these men live and find it to be 90 degrees, ranging from Cleveland to Bellington, Washington. But "western" is a relative term.

The same Journal has an article on the *Journal of the A.M.A.* which will interest all "stormy petrels" in the profession. We have no time to engage in the quarrel. The sharpest critic is often the best friend and it will not hurt the great national organ to show that it is adhering to a higher standard of ethics now than formerly, or that there is still room for improvement. In all kindness, we may say that the facts presented lead to a more charitable and lenient attitude on the part of the latter, toward independent journals which, in the struggle for existence cannot always follow the course that they would prefer. Then, too, many feel that a "judicial attitude" means giving the man with whom we differ a fair chance to present his own claims, and not a condemnation or a refusal of a hearing.

---

DR. GEORGE B. SHATTUCK who, for 31 years, has conducted the *Boston Medical and Surgical Journal,* has retired. We record the fact with mingled congratulations for his past work, regret at his leaving the field of active journalistic work, and best wishes for the future both of himself and of the Journal. The motto of the *Albany Medical Annals,* "Going out, they hand their lights to others" (or Greek words somewhat to that effect), is most appropriate on such an occasion. The *Boston Journal,* established 84 years ago this month, is one of the very few exchanges to which the BUFFALO JOURNAL has to show the deference due to seniority.

---

HARPER'S for December 1911, well illustrates the attraction of medicine for the public. Kipling has a story of obsessions due to the hereditary memory of maternal impressions. Mental and other disease is often worked into the plot of this author and, unfortunately, verisimilitude rather than verity, backed by his forceful pen, gives erroneous impressions. Max Sinclair contributes an amusing and nearly tragic story of the wiles of a Superintendent of Nurses who almost beguiled into matrimony the secretary of the hospital, via a suggested neurasthenia. Dr. Henry Smith Williams presents a really scientific article on the Struggle for Immunity.

